# Metaexoproteomics Reveals Microbial Behavior in the Ocean’s Interior

**DOI:** 10.3389/fmicb.2022.749874

**Published:** 2022-02-16

**Authors:** Zhang-Xian Xie, Yan-Bin He, Shu-Feng Zhang, Lin Lin, Ming-Hua Wang, Da-Zhi Wang

**Affiliations:** ^1^State Key Laboratory of Marine Environmental Science/College of the Environment and Ecology, Xiamen University, Xiamen, China; ^2^College of Ocean and Earth Sciences, Xiamen University, Xiamen, China; ^3^Southern Marine Science and Engineering Guangdong Laboratory (Zhuhai), Sun Yat-sen University, Zhuhai, China; ^4^BGI-Shenzhen, Shenzhen, China

**Keywords:** metaexoproteomics, exoprotein, microbial community, metabolic function, ocean water column

## Abstract

The proteins present in the extracellular environment of cells, named the “exoproteome,” are critical for microbial survival, growth, and interaction with their surroundings. However, little is known about microbial exoproteomes in natural marine environments. Here, we used a metaproteomic approach to characterize the exoprotein profiles (10 kDa-0.2 μm) throughout a water column in the South China Sea. Viruses, together with *Alpha-* and *Gammaproteobacteria* were the predominant contributors. However, the exoprotein-producing microbial communities varied with depth: SAR11 in the shallow waters, *Pseudomonadales* and *Nitrososphaeria* in the mesopelagic layer, and *Alteromonadales*, *Rhizobiales*, and *Betaproteobacteria* in the bathypelagic layer. Besides viral and unknown proteins, diverse transporters contributed substantially to the exoproteomes and varied vertically in their microbial origins, but presented similar patterns in their predicted substrate identities throughout the water column. Other microbial metabolic processes subject to vertical zonation included proteolysis, the oxidation of ammonia, nitrite and carbon monoxide, C1 metabolism, and the degradation of sulfur-containing dissolved organic matter (DOM). Our metaexoproteomic study provides insights into the depth-variable trends in the *in situ* ecological traits of the marine microbial community hidden in the non-cellular world, including nutrient cycling, niche partitioning and DOM remineralization.

## Introduction

Exoproteins are present in the extracellular environment in close proximity to specific biological systems after their release by cell secretion, lysis, or leakage (Christie-Oleza et al., 2015). These proteins are collectively designated the “exoproteome,” which is an indicator of the physiological state of the cells under specific conditions, and can provide insights into the interactions between microbial cells and their environments, including nutrient uptake and cell competition (Armengaud et al., 2012). Moreover, the significant contribution of exoproteins to the total extracellular enzymatic activity in marine ecosystem has been observed (Baltar et al., 2013, 2017). Cell-free active enzymes have also been documented in epipelagic and bathypelagic waters (Baltar, 2018). Therefore, exoproteome analyses, i.e., characterization of the composition, origin and function of exoproteins, are essential to fully understand the roles of microbes in natural ecosystems.

Several *in silico* analyses have shown that 8–44% of the encoded proteomes of marine bacteria can be exported to the extracellular environment as theoretical exoproteomes (Evans et al., 2007; Christie-Oleza et al., 2012, 2015), indicating the significance of exoproteins in marine microbial ecology. However, our knowledge of the exoproteomes of marine bacteria is still limited because we have lacked the appropriate methodology to study exoproteins. The shotgun proteomic approach has been used to study the exoproteome of a marine bacterium, *Pseudoalteromonas tunicata*, revealing the importance of iron transport and acquisition in this species (Evans et al., 2007). Using a similar approach, exoproteomic studies of two strains within the marine *Roseobacter* clade show unsurprisingly abundant repeat-in-toxin (RTX) proteins, which act as virulence factors in many pathogens (Christie-Oleza and Armengaud, 2010; Durighello et al., 2014). These studies demonstrate the feasibility of examining exoprotein profiles with this proteomic approach to investigate the ecological functions of marine microbes.

Exoprotein profiles not only differ among microbial groups across large phylogenetic distances, as shown in the studies cited above, but also among very closely related microbes. A comparative exoproteomic study of 12 *Roseobacter* strains shows that the RTX proteins are only overrepresented in some strains (Christie-Oleza et al., 2012). Another comparative exoproteomic study of eight *Synechococcus* strains reveals obvious variations in the exoprotein profiles of strains with similar genetic backgrounds (Christie-Oleza et al., 2015). Both studies also demonstrate that the exoproteomes of the same bacterial strains are growth-condition dependent. Therefore, given the microbial diversity and complex environments in natural marine habitats, the exoproteomes of marine environments should be very dynamic. However, to our knowledge, exoproteomic studies of marine microbes have been limited to two laboratory-cultured bacterial groups (Christie-Oleza et al., 2012, 2015), and little effort has been devoted to examine natural marine exoproteomes, in the discipline of “metaexoproteomics,” due to the lack of appropriate methodology to study complex environmental samples. Though, recent high-throughput analyses of metaproteomes in high molecular-weight dissolved organic matter (DOM) and marine viral concentrates from the euphotic zone have paved the way to the characterization of exoproteins in natural marine environments (Dong et al., 2013; Xie et al., 2018). Based on this method, a recent study is conducted on the exoproteomes collected from marine epipelagic to bathypelagic waters, but it only focuses on two extracellular enzyme groups, i.e., peptidases and carbohydrate-active enzymes (CAZymes) (Zhao et al., 2020).

The sunlit zone is the most productive ocean region, and this ecosystem relies greatly on energy metabolism fueled by light, e.g., photosynthesis by phytoplankton and photoherterotrophy by proteorhodopsin-containing bacteria (Ferrera et al., 2015). Below the sunlit layer, the environment moves progressively toward to the disappearance of light, lower temperatures, higher pressures, and a lower carbon supply, which largely determine the decline in biomass and the change in the composition of the microbiome (DeLong et al., 2006). Despite their energy supply based on nitrification and sulfur metabolism as well as diverse pathways of inorganic carbon fixation, microbes in the dark ocean are assumed to be primarily dependent on the heterotrophic metabolism of carbon exported from the sunlit layer (Swan et al., 2011; Pachiadaki et al., 2017; Acinas et al., 2021). The vertical stratification of oceanic microbes has been verified in the cellular fraction with both metagenomics and metatranscriptomics, e.g., the depth-variable metabolism of carbon, nitrogen and energy, and host-virus interactions (DeLong et al., 2006; Shi et al., 2010). However, whether there is similar vertical zonation in the cell-free fraction of the water column remains to be examined. Here, we present a metaexoproteomic analysis throughout the water column in the basin of the South China Sea (SCS), using a metaproteomic approach. We showed that the microbial contributors to and the functional categories of the vertical exoprotein profiles (10 kDa-0.2 μm) shifted from the sunlit zone to the dark deep ocean. The findings of this study shed light on the ecologically significant functions in the “non-cellular” world of the ocean.

## Materials and Methods

### *In situ* Sampling

Using a rosette sampler equipped with a 12 L Niskin Go-Flo bottle, metaexoproteomic samples were collected from the DCM (75 m), upper mesopelagic layer (200 m), and bathypelagic layer (3,000 m) at the SEATS station (18°N, 116°E) in the SCS in August 2012. To enrich the exoproteins for analysis, each sample was prepared from 100 L of seawater, and precautions were taken to avoid any possible contaminations during the experiment. In brief, sequential filtration with GF/F glass fiber filters (0.7 μm pore size; Whatman) and Durapore membrane filters (0.2 μm pore size, Millipore) was performed gently at a filtration rate of less than 1 L/min to eliminate any remaining cells once the seawater had arrived onboard. A final concentration of 0.01% (w/v) sodium dodecyl sulfate was added to increase protein recovery and 5 mM of sodium azide was added to inhibit microbial growth. A Pellicon 2 tangential flow filtration system equipped with a 10-kDa-cutoff regenerated cellulose membrane package (Millipore) was used to concentrate the filtrate to a volume of around 450 mL. The concentrates were stored at –80°C until a second concentration procedure was performed in the laboratory to obtain the final metaexoproteomic samples (around 10 mL). Cell counting of the filtrates using flow cytometry showed cell concentrations below the detection limit of flow cytometry (data not shown), indicating little contribution of intact cells to the samples. It should be noted that concentrating large volumes of seawater was a time-consuming, laborious and expensive process, especially with deep seawater. Therefore, we collected duplicate seawater samples from the DCM layer, but only one seawater sample each from the upper mesopelagic layer and the bathypelagic layer due to the limitation of ship time.

### Protein Extraction and Mass Spectrometry Analysis

The experimental procedures for protein precipitation, preparation and final trypsin digestion for the MS analysis were conducted as described previously (Xie et al., 2018). Briefly, the exoproteins were precipitated with ice-cold 20% trichloroacetic acid in acetone solution overnight at –20°C and were resuspended with rehydration buffer containing 7 M urea, 2 M thiourea, and 3-[(3-cholamidopropyl) dimethylammonium]-1-propane-sulfonate (4% w:v). Around 10–40 μg proteins of each sample were obtained and subjected to trypsin digestion (12 h, 1:20 enzyme to protein) twice after reduction with dithiothreitol and alkylation with iodoacetamide. The resulting peptide solution was fractionated with a Shimadzu LC-20AB HPLC system equipped with a Ultremex strong cation exchange column (4.6 × 250 mm, 5 μm particles; Phenomenex). The elution gradient was set as follows: 0–10 min, 100% buffer A [25 mM KH_2_PO_4_ in 25% acetonitrile (ACN), pH 3.0]; 10–40 min, 5–35% buffer B (25 mM KH_2_PO_4_, 1 M KCl in 25% ACN, pH 3.0); 40–41 min, 35–80% buffer B; 41–44 min, 80% buffer B. The peptide fractions were desalted with C18 columns and resuspended in buffer C [5% ACN, 0.1% formic acid (FA)]. The sample was loaded at 8 μL/min for 4 min, and analyzed under a elution gradient [0–40 min, 2–35% buffer D (95% ACN, 0.1% FA); 40–45 min, 35–80% buffer D; 45–49 min, 80% buffer D] at 300 nL/min on a Shimadzu LC-20AD nano-HPLC in line with a Thermo Q Exactive mass spectrometer using data-dependent fragmentation with a dynamic exclusion duration of 15 s. A normalized collision energy setting of 27.0 in the high-energy collision dissociation operating mode was used to fragment selected peptides. Resolutions of 70,000 and 17,500 for the Orbitrap analyzer were used for the MS and MS/MS scans, respectively, and the MS scans were set to between 350 and 2,000 Da.

### Protein Identification and Bioinformatic Analysis

The raw MS data for each sample were merged and formatted to MGF files with Proteome Discoverer (version 1.3.0.339; Thermo Fisher Scientific, San Jose, CA). Because we did not create sample-specific metagenomes, the proteins were identified and quantified with a two-step iterative strategy similar to the previously described MetaPro-IQ approach (Zhang et al., 2016). Briefly, more than 15 million protein-coding sequences were predicted from the vertical metagenomes of the SCS (sizes between 0.2 and 200 μm, and depths of 5 m, DCM, 200, 750, and 3,000 m) in a previous study (Chen et al., 2021), and combined with the global Ocean Microbial Reference Gene Catalog (OM-RGC, more than 40 million sequences) of marine viruses, prokaryotes, and picoeukaryotes (Sunagawa et al., 2015) to construct a non-redundant target-only database. The Mascot search engine (ver. 2.3.0; Matrix Science, London, United Kingdom) was used in the first-step search, and all matched protein sequences were extracted as the sample-specific database without any criteria. This reduced database was combined with the reverse decoy database and duplicate sequences were removed, thus a “combined non-redundant database” was created for the second step of the target-decoy search using the three search engines of MS-GF+, OMSSA, and X!Tandem integrated in the IPeak software (Wen et al., 2015). The search parameters were set as follows: precursor ion tolerance of 20 ppm; fragment ion tolerance of 0.02 Da; trypsin as the proteolytic enzyme, allowing for one missed cleavage; carbamidomethyl cysteine specified as a fixed modification and methionine oxidation (M) as a variable modification. In the second-step IPeak search, Percolator was used to re-score the peptide-spectrum matches from MS-GF+, OMSSA, and X!Tandem, and the results were combined based on the FDRScore algorithm at a false discovery rate (FDR) of 1%. Finally, a minimum of two peptides/proteins (at least one unique peptide) was used to accept the protein matches as identifications with high confidence. The highest scoring protein in the group matching the same peptide was selected as the representative protein.

A quantitative analysis was used to estimate the relative abundances of the proteins (P_*i*_) with the equation:


$$Pi=\frac{\frac{Si}{Li}}{\sum_{k=1}^{n}\left(\frac{Sk}{Lk}\right)}*100$$

in which the unique spectral counts (S_*i*_) are divided by the protein length (L_*i*_). The subscript i or k denotes a protein or a protein group identity, and n is the total number of proteins or protein groups detected in a sample.

All identified proteins were newly annotated using BLASTP against the Clusters of Orthologous Groups of proteins (COG) database,^1^ the Kyoto Encyclopedia of Genes and Genomes (KEGG) database (version 81), and the National Center for Biotechnology Information Non-redundant Protein database (NCBI-nr, 2017/9/24) with an *E*-value cutoff of 1e-5 and other default parameters. The taxonomic annotations were inferred from taxonomic information on the hits in the NCBI-nr database. Note that the taxonomic annotation of a bacterial protein was modified to that of a putative viral protein if the protein function was assigned to a viral structural protein.

## Results

### Overview of Exoproteomes

**FIGURE 1:**
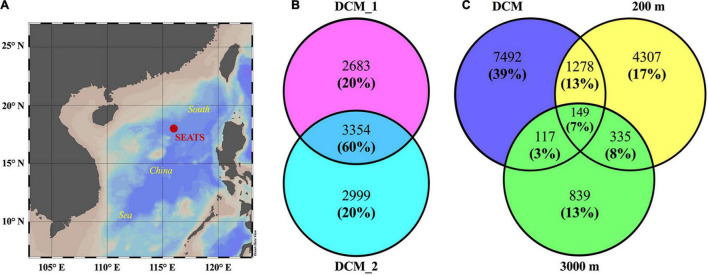
Numbers of proteins in the exoproteomes from the water layers at the deep chlorophyll maximum (DCM), 200 and 3,000 m at SEATS station in the South China Sea (SCS). The map **(A)** shows the position of sampling site. Venn diagrams show the number and relative abundance of exoproteomes between two biological replicates from the DCM **(B)** and between different water layers **(C)**. In Venn diagrams, numbers without and in brackets show the proteins counts of each part and their proportions of total relative abundance of four exoproteomes, respectively. Exoproteome from the DCM shown in **(C)** include all the non-redundant proteins identified in the two biological replicates.

A high-throughput shotgun metaproteomic approach was used to investigate exoprotein profiles collected from the deep chlorophyll maximum (DCM, 75 m), the upper mesopelagic layer (200 m), and the bathypelagic layer (3,000 m) in an oligotrophic basin of the SCS (Figure 1A). The tandem mass spectrometry (MS/MS) datasets generated were screened against a custom database containing environmental sequences from vertical metagenomes of the SCS (including one water column at the same site) and the currently largest marine gene reservoir (OM-RGC) with a two-step iterative strategy. They generated 36,367 unique peptides matching 65,251 unique spectra. With a stringent false discovery rate (FDR) cutoff of 1% and at least two matched peptides, 3,354 exoproteins, accounting for 60% of the protein abundance, were repeatedly detected in two biological replicates of the DCM sample (DCM_1 and DCM_2), indicating high reproducibility (Figure 1B). Finally, 6,037, 6,353, 6,069, and 1,440 exoproteins were identified in four samples, with a total output of 14,517 non-redundant exoproteins (Figure 1C and Supplementary Data Sheets 1–5). Among these non-redundant exoproteins, 1.8–9.8% occurred in at least two depth layers, whereas only 1.0% was detected at all depths, indicating the distinctness of the exoproteomes in each water layer (Figure 1C).

### Taxonomic Characterization of Exoproteomes

**FIGURE 2:**
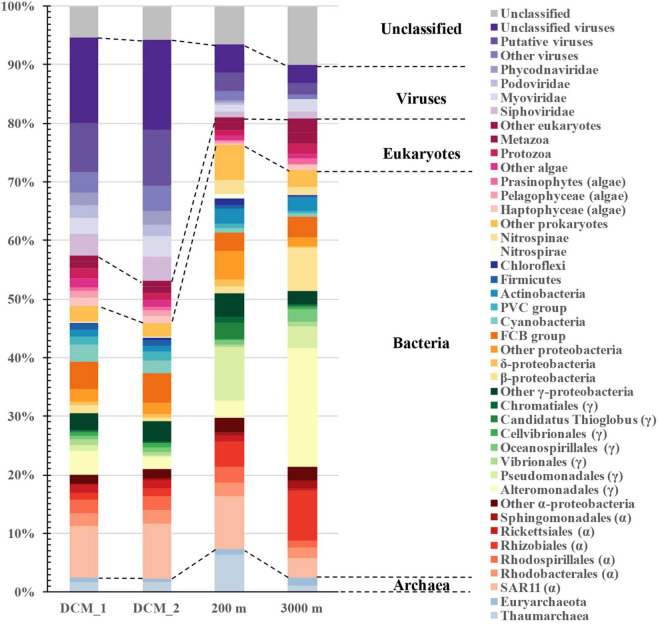
Distribution of vertical exoprotein profiles in the SCS in terms of their microbial origins. Data are represented as percentage of total relative abundance of each exoproteome.

The natural exoproteomes in the ocean contained exoproteins from all superkingdoms, although there was a small fraction of taxonomically unclassified proteins (Figure 2). Bacterial proteins dominated all the exoproteomes, and their relative abundances increased from 44% at the DCM to 69% in the dark bathypelagic layer. Most of them originated from *Proteobacteria* (30–58%), especially *Alpha-* and *Gammaproteobacteria*. Their descent taxonomic groups, including *Pelagibacterales* (SAR11), *Rhizobiales*, *Alteromonadales*, *Pseudomonadales*, and the FCB (*Fibrobacteres*, *Chlorobi*, and *Bacteroidetes*) group, contributed the most proteins to the exoproteomes. Viruses contributed similar amounts of protein (37–41%) as bacteria at the DCM, but the abundance of viral proteins decreased dramatically to 12% at 200 m and to 9% at 3,000 m. Except for the large amount of taxonomically unclassified proteins and putative viral proteins, most of the virus-associated proteins originated from the viral families *Siphoviridae* (1.1–4.2%), *Myoviridae* (1.0–3.5%), *Podoviridae* (0.02–2.1%), and *Phycodnaviridae* (0.07–2.2%). The abundances of eukaryotic proteins were similar among layers (4.7–8.9%), and the majority of them were assigned to eukaryotic phytoplankton, such as Haptophyceae (0.3–1.2%), Pelagophyceae (0.3–1.2%), and Prasinophyceae (0.2–0.8%). Archaea contributed the least proteins (2.3–7.6%) to the exoproteomes, and *Nitrososphaeria* [a reclassified class in GTDB database for the NCBI phylum *Thaumarchaeota* (Rinke et al., 2021), 1.0–6.3%] dominated this superkingdom.

The distribution patterns of microbial contributors to the exoproteomes varied throughout the water column. The protein abundances of most microbial groups declined with increasing depth. However, proteins from *Pseudomonadales* (9.1%), *Nitrososphaeria* (6.3%), *Candidatus* Thioglobus (2.6%), *Actinobacteria* (2.6%), *Nitrospinae* (2.3%), *Deltaproteobacteria* (1.1%), *Chloroflexi* (1.0%), *Nitrospirae* (1.0%), and *Chromatiales* (0.9%) were the most abundant at 200 m, whereas those from *Alteromonadales* (20.2%), *Rhizobiales* (8.6%), *Betaproteobacteria* (7.4%), *Oceanospirillales* (2.2%), and *Sphingomonadales* (1.3%) were particularly abundant at 3,000 m.

### Functional Characterization of Exoproteomes

Virus-associated proteins were one of the major components of the exoproteomes throughout the water column, although their abundance decreased from 41% at the DCM to 12% at 200 m and 9% at 3,000 m. Of the virus-associated proteins, 48–70% were viral structural components, such as capsid or tail proteins. The remaining virus-associated proteins were largely functionally uncharacterized (28–45%), with the exception of several virus-encoded auxiliary metabolic proteins (AMPs), including the photosystem II D1 and D2 proteins, chaperonin GroES, and transporters of iron complexes and amino acids (Supplementary Table 1).

**FIGURE 3:**
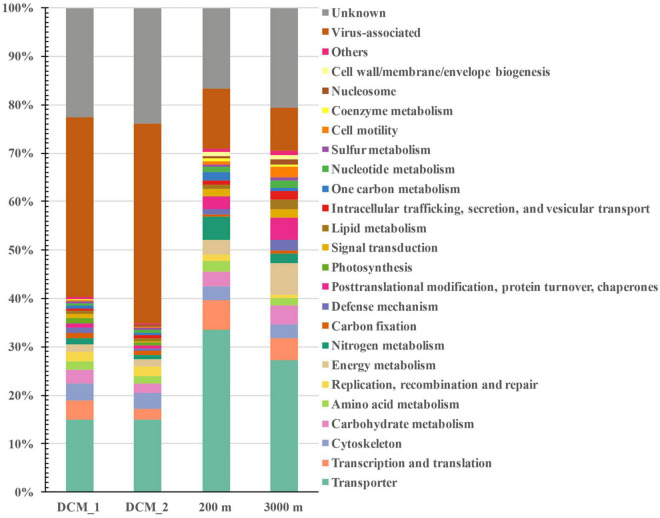
Distribution of vertical exoprotein profiles in the SCS in terms of their functional categories. Data are represented as percentage of total relative abundance of each exoproteome.

The non-viral proteins included cellular proteins with diverse functional categories (35–71%) and functionally unknown proteins (17–24%) (Figure 3). At all depths, transporters were the most abundant cellular proteins (15–34%), followed by moderately abundant proteins related to transcription and translation, energy metabolism, cytoskeleton, carbohydrate metabolism, posttranslational modification, protein turnover and chaperones, nitrogen metabolism, amino acid metabolism, defense mechanism, and cell motility. The remaining cellular proteins belonged to other functional categories and the abundance of each category did not exceed 2% in any exoproteome sample. The distributions of different functional categories were depth dependent. Proteins associated with cytoskeleton, replication, recombination and repair, carbon fixation, and photosynthesis were more abundant at the DCM than at the other two layers. However, proteins involved in transport, transcription and translation, nitrogen metabolism, amino acid metabolism, one-carbon metabolism, and coenzyme metabolism were most abundant at 200 m, and proteins relevant to posttranslational modification, protein turnover and chaperones, carbohydrate metabolism, lipid metabolism, and cell motility were most abundant at 3,000 m.

**FIGURE 4:**
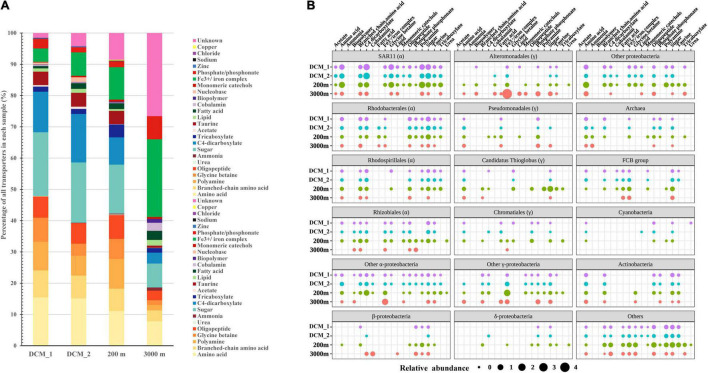
Distribution of transporters in the vertical exoproteomes from the SCS. **(A)** Transporter profiles classified by their predicted substrates. Data are represented as percentage of total relative abundance of transporters detected in each sample. **(B)** Vertical profiles of transporters from different taxa. Bubble size indicates the relative abundance of transporters summed by category.

**FIGURE 5:**
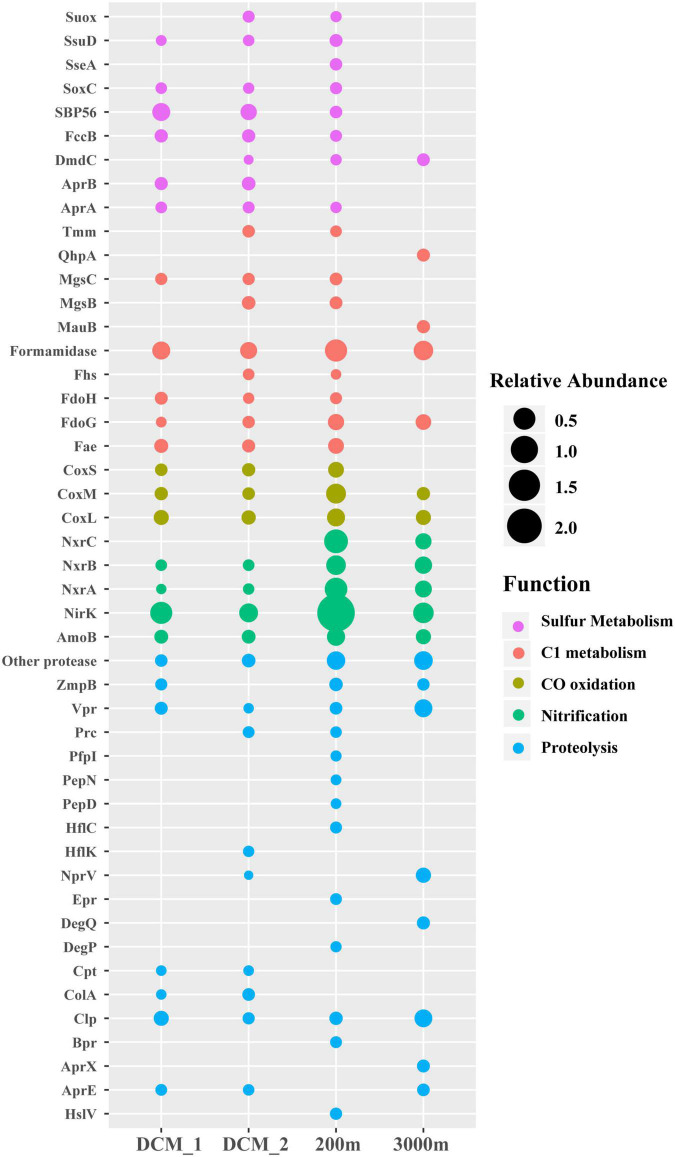
Depth-variable pattern of selected exoproteins detected in the vertical exoproteomes in the SCS. Bubble size indicates the relative abundance of each protein. Suox, sulfite oxidase; SsuD, alkanesulfonate monooxygenase; SseA, thiosulfate/3-mercaptopyruvate sulfurtransferase; SoxC, sulfane dehydrogenase subunit SoxC; SBP56, 56-kDa selenium-binding protein; FccB, sulfide dehydrogenase; DmdC, 3-(methylthio) propanoyl-CoA dehydrogenase; AprA and AprB, adenylylsulfate reductase subunits A and B; Tmm, trimethylamine monooxygenase; QhpA, quinohemoprotein amine dehydrogenase; MgsB and MgsC, methylamine-glutamate N-methyltransferase subunits B and C; MauB, methylamine dehydrogenase heavy chain; Fhs, formate-tetrahydrofolate ligase; FdoH, formate dehydrogenase iron-sulfur subunit; FdoG, formate dehydrogenase major subunit; Fae, 5,6,7,8-tetrahydromethanopterin hydro-lyase; CoxL, CoxM, and CoxS, large, medium and small subunits of aerobic carbon-monoxide dehydrogenase; NxrA, NxrB, and NxrC, alpha, beta and gamma subunits of nitrite oxidoreductase; NirK, nitrite reductase (NO-forming); AmoB, ammonia monooxygenase subunit B; ZmpB, zinc metalloprotease ZmpB; Vpr, minor extracellular serine protease Vpr; Prc, carboxyl-terminal processing protease; PepN, aminopeptidase N; PfpI, protease I; PepD, putative serine protease PepD; HflK and HflC, membrane protease subunits HflK and HflC; NprV, vibriolysin; Epr, minor extracellular protease Epr; DegQ, serine protease DegQ; DegP, serine protease Do; Cpt, carboxypeptidase T; ColA, microbial collagenase; Clp, ATP-dependent Clp protease; Bpr, bacillopeptidase F; AprX, serine protease AprX; AprE, subtilisin; HslV, ATP-dependent HslUV protease, peptidase subunit HslV.

Many non-viral exoproteins were found to be related to nutrient cycling and other important biogeochemical processes mediated by bacteria and archaea (Figures 4, 5). The distribution of transporters in the exoproteomes throughout the water column of the SCS provided the information on the substrate uptake in exoprotein-producing microbial communities at depths (Figure 4A). The substrate prediction was conducted based on the KEGG annotation. With the exception of urea, nucleobases, and inorganic ions, almost all the known substrates associated with transporters were detected in each sample (Figure 4A). Transporters indicating the utilization of fatty acids, lipids, ammonia, and phosphate/phosphonate were more frequently detected in the dark ocean than at the DCM. Transporters of Fe^3+^ and iron complexes were abundantly detected throughout the water column and were the dominant transporters at 3,000 m, suggesting that iron was one of the most important nutrients recycled in the deep ocean. Vertical variations in transporters, classified by predicted substrates, were apparent among the different prokaryotic taxonomic groups (Figure 4B). The substrate uptake pattern of the exoprotein-producing community at 200 m was rather similar to that at the DCM. One small difference was that several microbial groups, including *Gammaproteobacteria*, *Betaproteobacteria*, *Deltaproteobacteria*, *Rhizobiales*, and *Archaea*, consumed more diverse substrates at 200 m. However, the patterns at the DCM and at 3,000 m diverged in the microbial origins of transporters (Figure 4B). For example, only a small fraction of the transporters from many microbial groups still remained at 3,000 m, and more substrates were associated with *Alteromonadales* and *Betaproteobacteria* at 3,000 m than at the DCM. Moreover, although exoproteins involved in proteolysis, nitrification, the oxidation of carbon monoxide, C1 metabolism and sulfur metabolism were less abundant than transporters, variations within these functional categories were also observed along the water column (Figure 5).

Large amounts of non-viral exoproteins were from eukaryotes (4.7–8.9%) and were associated with diverse functions. In the 3,000 m layer, 30, 17, 14, and 8% of eukaryotic exoproteins were associated with cytoskeleton, protein chaperones, translation and ribosomal structure, and energy metabolism, respectively. More than one third of these exoproteins were from eukaryotic phytoplankton, and included actin, tubulin, heat shock proteins, ribosomal proteins, translation factors, and ATPases. Interestingly, most of these exoproteins from eukaryotic phytoplankton, including the rubisco large subunit (rbcL), were also detected in the upper two layers, providing the first exoprotein evidence of biological pump in the ocean.

## Discussion

### Vertical Changes in the Exoprotein-Producing Microbial Community

Exoproteomic studies of laboratory-cultured marine bacteria, such as *Roseobacter* and *Synechococcus*, have been reported (Christie-Oleza and Armengaud, 2010; Christie-Oleza et al., 2012, 2015; Durighello et al., 2014). However, little is known about the exoproteomics in natural marine environments. In this study, we used a metaproteomic approach to investigate the exoproteomes throughout the water column. Diverse microbes contributed to the exoproteomes at each layer and the origins of the exoproteins in the seawater were highly complex. Most of the microbial groups commonly found in the global ocean contributed to the marine exoproteomes. However, the relative abundances of the exoproteins varied in each layer in terms of their microbial contributors. This pattern can be partly explained by the genetic variations among different microbial groups. For example, theoretically, 30% of the genes in the *Roseobacter* genome encode exoproteins, whereas this increases to 40% in both cyanobacteria and SAR11 (Christie-Oleza et al., 2012, 2015). The differences in biomass among microbial groups was another important factor affecting the exoprotein abundances. SAR11 and cyanobacteria, encoding similar proportions of exoproteome proteins, are both abundant at the DCM in the SCS (Zhang et al., 2014), which is consistent with their highly abundant exoproteins (Figure 2). The physiological status of cells or the contents released from dead cells can also change the exoproteomic outcomes (Christie-Oleza et al., 2015), and the effect of these factors cannot be excluded. For example, the exoprotein abundance of hosts (e.g., cyanobacteria and SAR11 bacteria) increased with that of their associated viruses (Supplementary Figure 1), suggesting that viral infection affects the exoprotein status. Moreover, microbes associated with sinking particles might change physiologically or die in the deeper waters, consequently releasing their cellular contents into the extracellular milieu. Overall, the exoprotein profiles of the microbial communities might be associated with the diverse responses of different microbes to their environments.

The exoprotein-producing microbial community changed from the sunlit zone to the dark deep ocean (Figure 2). To some extent, the exoprotein abundance of many bacterial groups along the water column showed similar trends with the vertical distribution of the bacterial population based on 16S rRNA genes abundances in the SCS (Zhang et al., 2014). These microbial groups included SAR11, *Rhizobiales*, *Sphingomonadales*, *Alteromonadales*, *Rhodobacterales*, *Cyanobacteria*, *Nitrospinae*, and the FCB group. The highly abundant SAR11 exoproteins in each layer were consistent with the wide distribution of these dominant bacteria in the global ocean (Morris et al., 2002). *Alteromonadales*, *Betaproteobacteria*, and *Oceanospirillales* are among the abundant bacterial groups in the deep-sea microbial community (Eloe et al., 2011; Salazar et al., 2016) and contributed strongly to the nonviral exoproteins at 3,000 m (Figure 2). However, the exoproteins of *Pseudomonadales* and *Candidatus* Thioglobus were especially abundant in the mesopelagic layer (Figure 2). Overall, the pattern of exoprotein abundances in the water column was related to the distributions of the microbes in which they originated, somehow reflecting microbial niche partitioning along the water column.

Eukaryotic phytoplankton contributed more than half the eukaryotic exoproteins at the DCM, but declined to one-third in the other two layers, consistent with the vertical distribution of phytoplankton in the ocean. The abundances of exoproteins derived from their predators, such as protozoa and *Phycodnaviridae*, also declined throughout the water column. This observation, together with the identification of cytoplasmic proteins such as ribosomal proteins, translation factors and rbcL from eukaryotic phytoplankton, led us to postulate that the lysis of phytoplankton cells by their predators was one of the mechanisms by which the exoproteins of phytoplankton were produced in marine environments. Several studies have reported proteins from eukaryotic phytoplankton in size fractions both above and below 0.2 μm beneath the euphotic zone (Dong et al., 2010; Wang et al., 2011; Moore et al., 2012). The observation of phytoplankton proteins in these studies could be attributed to preserved proteinaceous materials that have sunk from the euphotic zone, where phytoplankton usually live. Protein solubilized from sinking particles or released from live phytoplankton cells may result in the presence of exoproteins from surface phytoplankton in aphotic waters. Viable phytoplankton cells from the surface ocean have been observed in the deep ocean (Lochte and Turley, 1988; Jiao et al., 2013; Xu et al., 2018). Consistent with findings in the deep Western Pacific Ocean (Xu et al., 2018), the presence of eukaryotic exoproteins in the deep SCS supports the notion that *Haptophyceae*, *Pelagophyceae*, and *Prasinophyceae* are the major eukaryotic phytoplankton contributing to the biological carbon pump that fuels the carbon pool in the deep sea.

Archaea are abundant below the euphotic zone (Karner et al., 2001). In the present study, the higher abundance of archaeal exoproteins at 200 m than at the DCM supports the idea that the dark ocean is the preferred habitat of archaea. In the ocean, *Nitrososphaeria* often dominate *Euryarchaeota* (Karner et al., 2001; Salazar et al., 2016). Consistent with their greater biomass, more exoproteins of *Nitrososphaeria* than *Euryarchaeota* were observed, especially at 200 m. Together, these data emphasize the ecological importance of *Nitrososphaeria* within the archaea, particularly in the dark ocean.

### Vertical Changes in Microbial Functions

#### Nutrient Uptake

Among the functional cellular proteins detected, transporters were the most abundant category in each layer (Figure 3). This is consistent with the high abundance of transporters in both the theoretical and experimental exoproteomes of laboratory-cultured marine bacteria (Christie-Oleza and Armengaud, 2010; Armengaud et al., 2012; Christie-Oleza et al., 2012, 2015). Most transporters detected in this study were the periplasmic components of transport systems or outer-membrane receptor proteins, which are exported to the extracellular space as the prime determinants of the selectivity of substrate identities. The presence of these highly abundant transporters indicated that one of the important ecological functions of the microbial exoproteome was closely associated with nutrient scavenging. The highly abundant transporters in the deeper waters suggested that nutrient-associated interactions between microbes and their surroundings also occurred in the dark ocean.

The variability in transporters in terms of their predicted substrates and phylogenetic origins provides insights into the nutrient utilization dynamics of the microbial communities in the ocean (Sowell et al., 2009, 2011; Morris et al., 2010; Williams et al., 2012; Georges et al., 2014; Bergauer et al., 2018; Xie et al., 2018). The predicted substrate identities of the transporters in the vertical exoproteomes suggested that amino acids (including branched-chain amino acids), polyamines, glycine betaine, oligopeptides, taurine, carbohydrates, and carboxylates were the major components of the labile dissolved organic nutrients utilized by microbes throughout the water column in the SCS. These results paralleled the results of a metaproteomic study in the Atlantic Ocean that targeted the microbial community in the size fraction between 0.2 and 0.8 μm (Bergauer et al., 2018).

The predicted substrates of the transporters in the exoproteomes in each layer were linked to the microbial compositions of the communities (Figure 4B). These transporters mainly originated from lineages of *Alphaproteobacteria*, *Gammaproteobacteria*, *Betaproteobacteria*, the FCB group, *Actinobacteria*, and *Archaea*, consistent with the observations in other metaproteomic studies in the cellular size fraction (Morris et al., 2010; Bergauer et al., 2018). SAR11, *Rhodobacterales*, *Rhodospirillales*, and *Rhizobiales* were strongly representative *Alphaproteobacteria* in the shallow layers but contributed less in the deep sea; *Candidatus* Thioglobus, *Chromatiales*, the FCB group, *Actinobacteria*, and *Archaea* contributed more-abundant transporters at 200 m than in the other two layers; whereas *Alteromonadales*, *Pseudomonadales*, and *Betaproteobacteria* contributed more transporters with increasing depth. Therefore, it appears that divergent microbial groups drive nutrient cycling at different depths. As in a previous study (Bergauer et al., 2018), the taxa associated with substrate-classified transporters changed throughout the oceanic water column (Figure 4B). Based on the transporter results for different size fractions, both studies suggest the vertical stratification of nutrient-based niche partitioning in the ocean.

Overall, the transporters abundantly present in the vertical exoproteomes lent support to the depth-dependent variation in microbe-nutrient interactions in the ocean. Although the microbial origins of the transporters changed throughout the water column, the substrates utilized by the communities were largely labile DOM and changed only slightly with depth, which is similar to the results of a previous metaproteomic study (Bergauer et al., 2018). This, together with the detection of exoproteins from surface phytoplankton at 3,000 m, also supported the hypothesis that deep sea microbes largely relied on the DOM solubilized from sinking particles, rather than on its local recalcitrant counterpart (Bergauer et al., 2018).

#### Proteolysis

Proteases and peptidases are a class of enzymes that break proteins into smaller polypeptides or single amino acids. They are frequently detected in the exoproteomes of marine bacterial isolates (Christie-Oleza et al., 2012, 2015) and the microbial community in the ocean (Zhao et al., 2020), and at least 20 different proteases were identified in this study (Figure 5), demonstrating that proteases are a common component of marine exoproteomes (Armengaud et al., 2012). The total abundance of all proteases increased from epipelagic to bathypelagic waters in the SCS, and this pattern is consistent with those in other ocean regions reported in a previous exoproteomic study (Zhao et al., 2020), suggesting that protein recycling became increasingly important as a pathway of carbon and nitrogen supply for the microbial community in deeper waters. However, different proteases varied in their vertical distributions, including the extracellular proteases Vpr, Epr, Bpr, AprE/X, and NprV. The Vpr protease increased in abundance with depth. Epr and Bpr were only detected at 200 m, where AprE/X and NprV were undetected, and AprE/X and NprV were most abundant at 3,000 m. These five proteases act specifically in proteolysis, but may also have other functions. For example, the secretion of Epr is involved in bacterial swarming motility (Murudkar et al., 2006). Vertical variations in proteases in terms of the enzyme species and their abundances probably reflect the changes in the protein substrates available for degradation and other protease-mediated processes at different depths.

#### Nitrification

A protein involved in the first step in nitrification, ammonia monooxygenase subunit B (AmoB) from *Nitrososphaeria* was detected in each layer. The failure to detect other subunits of Amo is consistent with the presence of abundant AmoB, as has been observed in the proteomes of laboratory-cultured isolates and natural ammonia-oxidizing archaea (AOA) populations (Georges et al., 2014; Hawley et al., 2014; Santoro et al., 2015; Kerou et al., 2016; Qin et al., 2017). Moreover, protein homologues of the NO-forming nitrite reductase (NirK) family detected here all originated from *Nitrososphaeria*. In a previous model of thaumarchaeal ammonia oxidation, NirK reduced nitrite to nitric oxide (NO), which was subsequently used for hydroxylamine oxidation, although this was not supported by isotopic trace experiments (Santoro et al., 2011; Kozlowski et al., 2016). Instead, in new models, NirK is proposed to act in the two-step oxidation of hydroxylamine to NO and then to nitrite (Carini et al., 2018). Regardless of this debate, both Amo and NirK are believed to be the core components of the ammonia oxidation machinery. In the model, the predicted location of these proteins is the thaumarchaeal pseudoperiplasm (Carini et al., 2018), which is consistent with their detection in exoproteomes. NirK was more abundant than AmoB, consistent with the higher expression of NirK transcripts than AmoB transcripts (Lund et al., 2012). Similar to a previous study (Carini et al., 2018), only ammonium transporters from *Nitrososphaeria* were coexpressed with AmoB and NirK in the present study, and were more abundantly represented in the aphotic zone, especially at 200 m (Figure 5). Ammonia oxidation was mainly associated with *Nitrososphaeria* and was more abundantly present below the bottom of euphotic zone in the SCS. In addition, an energy link between carbon fixation and ammonia oxidation in AOA has been proposed (Williams et al., 2012; Georges et al., 2014; Santoro et al., 2015). Consistent with this, proteins of *Nitrososphaeria* involved in carbon fixation in the hydroxypropionate-hydroxybutylate cycle were identified, including acetyl-/propionyl-CoA carboxylase, 4-hydroxybutyryl-CoA dehydratase, 3-hydroxypropionyl-CoA dehydratase, and methylmalonyl-CoA mutase.

Nearly all nitrite oxidoreductases (NXRs) were associated with two groups of nitrite-oxidizing bacteria (NOB), *Nitrospinae* and *Nitrospirae*, consistent with the genetic capacity of their cultured isolates and natural lineages for nitrite oxidation within the nitrification process (Lücker et al., 2010; Lucker et al., 2013; Koch et al., 2015; Pachiadaki et al., 2017). All subunits of NXR (NxrABC) showed the same vertical distribution pattern as Amo and NirK. This close co-occurrence relationship might support the previously proposed reciprocal feeding between AOA and NOB (Lücker et al., 2010; Pachiadaki et al., 2017). NXR proteins of *Nitrospirae* were only detected at 200 m, and were only half as abundant as their *Nitrospinae* counterparts, consistent with the previous observation of their transcripts in the SCS (Zhang et al., 2020). The predominance of *Nitrospinae* over *Nitrospirae* in the ocean in terms of exoprotein abundance is also consistent with observations based on a metagenomic or metatranscriptomic recruitment analysis (Pachiadaki et al., 2017; Zhang et al., 2020). Taken together, these data indicated that the nitrification in the water column of the SCS was achieved via ammonia oxidation by AOA and nitrite oxidation by NOB, and occurred most actively immediately below the euphotic zone.

#### Carbon Monoxide Oxidation and C1 Metabolism

A wide range of subunits (CoxL, CoxM, and CoxS) of aerobic carbon monoxide dehydrogenase (CODH), which oxidizes CO to CO_2_, were detected from diverse heterotrophic bacteria, including SAR11, *Bacteroidetes*, *Actinobacteria*, and *Chloroflexi*, indicating that they probably utilize CO. It has been estimated that *CoxL* genes are as abundant as one per 14 bacterial cells, based on a marine metagenomic library (Moran et al., 2004). Therefore, the frequent detection of CODH proteins throughout the water column in this and other studies (Georges et al., 2014) demonstrated the ecological significance of CO oxidation in the ocean. It has been reported that up to 88% of CO is oxidized by microbes, thus greatly reducing the entry of this greenhouse gas into the atmosphere (Tolli and Taylor, 2005). Therefore, the detection of CODH confirms that bacterial CO oxidation was an important mechanism by which CO sinks in the SCS.

It has been experimentally demonstrated that SAR11 are versatile in the utilization of diverse C1 or methylated compounds, mainly for energy production rather than carbon assimilation, in a process called “carboxidovory” (Sun et al., 2011). Proteins associated with the relevant metabolic processes were identified from SAR11, including Tmm, which is involved in the conversion of trimethylamine (TMA) to trimethylamine N-oxide; MgsB and MgsC, which oxidizes methylamine to formaldehyde; Fae, which converts formaldehyde to formate via the tetrahydromethanopterin pathway; formamidase, which degrades formamide to formate; and Fhs, which participates in the assimilation of formate via the tetrahydrofolate pathway (Sun et al., 2011, 2019; Martinez-Gomez et al., 2013). This supported the utilization of TMA, methylamine, formaldehyde, formamide, and formate by SAR11. Interestingly, no one-step formate oxidation to CO_2_ occurred in SAR11 because no formate dehydrogenase was detected from SAR11, but only from other bacteria. In addition, methylamine was probably not oxidized by SAR11 but degraded by *Alteromonas* at 3,000 m, because the MauB and QhpA proteins in another methylamine oxidation pathway were detected. The vertical distribution of these proteins indicated that the roles of SAR11 in the cycling of low-molecular-weight DOM are depth-dependent.

#### Sulfur Metabolism

Notably, a suite of proteins involved in sulfur metabolism were detected, including DmdC, which acts in the pathway of dimethylsulfoniopropionate demethylation; SBP56, which acts in the oxidation of methanethiol; and ssuD, which acts in the degradation of alkanesulfonate (Sun et al., 2016; Eyice et al., 2017). Except for DmdC, these proteins were not found in the exoproteome at 3,000 m, indicating that the upper layers of the SCS were the main regions of remineralization of these sulfur-containing DOMs. Interestingly, adenylylsulfate reductase and sulfite oxidase predominantly originated from SAR11, and are thought to detoxify sulfite, a byproduct of taurine degradation, in this bacterial group (Williams et al., 2012). Similar to the distribution of these proteins, SAR11 transporters that acted in taurine uptake were only found at the DCM and 200 m, indicating that the SAR11-mediated remineralization of taurine was probably confined to shallow waters. We also noticed that exoprotein-linked sulfur metabolism in the deep SCS was not as abundant as that in a recent metagenomic study of the global dark ocean (Acinas et al., 2021). This might be attributable to differences in the regulation of gene expression and the release mechanisms of these proteins at different locations.

#### Host-Virus Interaction

The significant contribution of viral structural proteins to the metaexoproteomes throughout the water column demonstrated the presence of abundant viruses in the ocean and indicated the potential interactions between viruses and their hosts. The presence of and variations of host-virus interaction at depths were verified with the detection of viral AMPs in the exoproteomes (Supplementary Table 1). AMPs are thought to be expressed to reprogram the host metabolism during viral infection (Lindell et al., 2005). Therefore, despite that our metaproteomics-based approach could only detected known viral AMPs in the public databases, these AMPs present in the cell-free fraction probably derive from the recent release of these proteins from virus-infected cells. Furthermore, the prevalence and expression of the viral *psbA* gene in the surface ocean (Sharon et al., 2007) and the rapid sinking of virus-infected cells (Suttle, 2005) have been reported. Our metaexoproteomic study not only detected viral psbA proteins in both layers of the DCM and 200 m, but also identified a psbD protein from *Synechococcus* phage in the 3,000 m layer, suggesting that some cyanobacterial cells in the surface ocean were probably phage-infected and were lysed as they sank to the deep ocean. Overall, our results demonstrate the depth-dependence of host-virus interactions and their roles in carbon export in the ocean.

## Conclusion

In this metaexoproteomic analysis, we investigate the natural exoprotein profiles throughout an oceanic water column. Our study sheds light on the complex origins of the exoproteins and the predominant contributors to the exoproteomes, such as viruses, *Alpha*- and *Gammaproteobacteria*, from the sunlit zone to the deep dark ocean. The origin of exoproteins suggests that these microbial groups make a significant contribution to the oceanic DOM pool. However, the contribution of abundant taxa is depth-dependent, e.g., SAR11 in the shallow waters, *Pseudomonadales* and *Nitrososphaeria* in the mesopelagic layer, and *Alteromonadales*, *Rhizobiales* and *Betaproteobacteria* in the bathypelagic layer. Furthermore, we demonstrate the vertical zonation of exoprotein-linked functions, including substrate transport, protein degradation, nitrification, oxidation of C1 and methylated compounds, sulfur metabolism and host-virus interaction. Especially, microbial players are depth-dependent despite of utilizing similar DOM substrates; different extracellular proteases are involved in protein recycling at different layers; nitrification by AOA and NOB is more abundantly present below the euphotic zone, whereas degradation of sulfur-containing DOMs and host-virus interactions are more frequently observed in the shallow layers. The depth-variable trends in these categories of metabolic functions provide new insights into the dynamic interactions between microbes and their surroundings in terms of nutrient uptake, DOM remineralization, and niche partitioning in the ocean.

Cell-free active enzymes have been documented in epipelagic and bathypelagic waters, and their activities are regarded as the “gatekeepers” of biogeochemical cycles (Baltar, 2018). However, our current knowledge is mainly based on a few extracellular enzymes. Our study indicates the presence of novel exoproteins with potential connections to diverse biogeochemical processes in the ocean. In future metaexoproteomic studies, more efforts should be directed toward the kinds of exoproteins that are functionally active in the noncellular world, with the integration of multiple meta-omic approaches, such as metaexoproteomics and metabonomics, as well as *in situ* metabolic rate measurements. This will comprehensively improve our understanding of microbial functions and roles in the ocean.

It should be pointed out that only one sample was collected in both the meso- and bathypelagic layers owing to the limitation of sampling time. Because the content of protein in particulate organic carbon is very low, large volumes of seawater must be concentrated to obtain sufficient protein for proteomic analysis, which is a time-consuming, laborious and expensive process. This brings forward challenges for repeated sampling, especially in deep waters. In oceanography, oceanographic consistency across the vertical depth structure is a good substitute for biological replicates when validating results, because the fluid feature of the ocean may allow different water masses to be captured, even within a short time, leading to unreal variations in the microbial community and other properties among replicates (Saito et al., 2019). The strategy of sampling more sites can also improve the results, as demonstrated in the previous study of oceanic exoproteomes focusing on CAZymes and peptidases (Zhao et al., 2020). Overall, different sampling strategies should be adopted according to actual field conditions, to avoid the analytical errors.

## Data Availability Statement

The data presented in the study are deposited in the ProteomeXchange repository available here: www.ebi.ac.uk/pride/archive/, accession number PXD020896.

## Author Contributions

D-ZW and Z-XX conceived the study. Z-XX, S-FZ, and M-HW collected the samples. Z-XX and Y-BH analyzed the data. Z-XX and LL performed the proteomic experiment. Z-XX, Y-BH, and D-ZW wrote the manuscript. All authors contributed to the discussion of the results.

## Conflict of Interest

The authors declare that the research was conducted in the absence of any commercial or financial relationships that could be construed as a potential conflict of interest.

## Publisher’s Note

All claims expressed in this article are solely those of the authors and do not necessarily represent those of their affiliated organizations, or those of the publisher, the editors and the reviewers. Any product that may be evaluated in this article, or claim that may be made by its manufacturer, is not guaranteed or endorsed by the publisher.

## Footnotes

1
ftp.ncbi.nih.gov/pub/COG/COG2014/data

## Supplementary Material

Supplementary Figure 1 Scatter plot showing the abundance relationship between phage proteins and host exoproteins. Dots with different colors indicate different host groups.

Supplementary Data Sheet 1 Sequence information for exoproteins identified in the DCM_1 sample.

Supplementary Data Sheet 2 Sequence information for exoproteins identified in the DCM_2 sample.

Supplementary Data Sheet 3 Sequence information for exoproteins identified in the 200 m sample.

Supplementary Data Sheet 4 Sequence information for exoproteins identified in the 3000 m sample.

Supplementary Data Sheet 5 Sequence information for non-redundant exoproteins identified in all samples.

## References

[B1] Acinas S. G. Sánchez P. Salazar G. Cornejo-Castillo F. M. Sebastián M. Logares R. (2021). Deep ocean metagenomes provide insight into the metabolic architecture of bathypelagic microbial communities. *Commun. Biol.* 4:604. 10.1038/s42003-021-02112-2 34021239 PMC8139981

[B2] Armengaud J. Christie-Oleza J. A. Clair G. Malard V. Duport C. (2012). Exoproteomics: exploring the world around biological systems. *Expert Rev. Proteomics* 9 561–575. 10.1586/epr.12.52 23194272

[B3] Baltar F. (2018). Watch out for the “living dead”: cell-free enzymes and their fate. *Front. Microbiol.* 8:2438. 10.3389/fmicb.2017.02438 29354095 PMC5758490

[B4] Baltar F. Aristegui J. Gasol J. M. Yokokawa T. Herndl G. J. (2013). Bacterial versus archaeal origin of extracellular enzymatic activity in the Northeast Atlantic deep waters. *Microb. Ecol.* 65 277–288. 10.1007/s00248-012-0126-7 23015014

[B5] Baltar F. Morán X. A. G. Lønborg C. (2017). Warming and organic matter sources impact the proportion of dissolved to total activities in marine extracellular enzymatic rates. *Biogeochemistry* 133 307–316. 10.1007/s10533-017-0334-9

[B6] Bergauer K. Fernandez-Guerra A. Garcia J. A. L. Sprenger R. R. Stepanauskas R. Pachiadaki M. G. (2018). Organic matter processing by microbial communities throughout the Atlantic water column as revealed by metaproteomics. *Proc. Natl. Acad. Sci. U.S.A.* 115 E400–E408. 10.1073/pnas.1708779115 29255014 PMC5776962

[B7] Carini P. Dupont C. L. Santoro A. E. (2018). Patterns of thaumarchaeal gene expression in culture and diverse marine environments. *Environ. Microbiol.* 20 2112–2124. 29626379 10.1111/1462-2920.14107

[B8] Chen S. He Y.-B. Xie Z.-X. Kong L.-F. Yan K.-Q. Li D.-X. (2021). Metaproteomics reveals nutrient availability shaping distinct microbial community and metabolic niche in the nutrient-depleted and replete layers of an oligotrophic euphotic zone. *Sci. Total Environ.* 774:145123. 10.1016/j.scitotenv.2021.145123

[B9] Christie-Oleza J. A. Armengaud J. (2010). In-depth analysis of exoproteomes from marine bacteria by shotgun liquid chromatography-tandem mass spectrometry: the *Ruegeria pomeroyi* DSS-3 case-study. *Mar. Drugs* 8 2223–2239. 10.3390/md8082223 20948905 PMC2953401

[B10] Christie-Oleza J. A. Armengaud J. Guerin P. Scanlan D. J. (2015). Functional distinctness in the exoproteomes of marine *Synechococcus*. *Environ. Microbiol.* 17 3781–3794. 10.1111/1462-2920.12822 25727668 PMC4949707

[B11] Christie-Oleza J. A. Pina-Villalonga J. M. Bosch R. Nogales B. Armengaud J. (2012). Comparative proteogenomics of twelve *Roseobacter* exoproteomes reveals different adaptive strategies among these marine bacteria. *Mol. Cell. Proteomics* 11:M111.013110. 10.1074/mcp.M111.013110 22122883 PMC3277765

[B12] DeLong E. F. Preston C. M. Mincer T. Rich V. Hallam S. J. Frigaard N. U. (2006). Community genomics among stratified microbial assemblages in the ocean’s interior. *Science* 311 496–503. 10.1126/science.1120250 16439655

[B13] Dong H.-P. Wang D.-Z. Dai M. Hong H.-S. (2010). Characterization of particulate organic matter in the water column of the South China Sea using a shotgun proteomic approach. *Limnol. Oceanogr.* 55 1565–1578. 10.4319/lo.2010.55.4.1565

[B14] Dong H.-P. Wang D.-Z. Xie Z.-X. Dai M.-H. Hong H.-S. (2013). Metaproteomic characterization of high molecular weight dissolved organic matter in surface seawaters in the South China Sea. *Geochim. Cosmochim. Acta* 109 51–61. 10.1016/j.gca.2013.01.041

[B15] Durighello E. Christie-Oleza J. A. Armengaud J. (2014). Assessing the exoproteome of marine bacteria, lesson from a RTX-toxin abundantly secreted by *Phaeobacter* strain DSM 17395. *PLoS One* 9:e89691. 10.1371/journal.pone.0089691 24586966 PMC3933643

[B16] Eloe E. A. Shulse C. N. Fadrosh D. W. Williamson S. J. Allen E. E. Bartlett D. H. (2011). Compositional differences in particle-associated and free-living microbial assemblages from an extreme deep-ocean environment. *Environ. Microbiol. Rep.* 3 449–458. 10.1111/j.1758-2229.2010.00223.x 23761307

[B17] Evans F. F. Raftery M. J. Egan S. Kjelleberg S. (2007). Profiling the secretome of the marine bacterium *Pseudoalteromonas tunicata* using amine-specific isobaric tagging (iTRAQ). *J. Proteome Res.* 6 967–975. 10.1021/pr060416x 17330939

[B18] Eyice Ö Myronova N. Pol A. Carrión O. Todd J. D. Smith T. J. (2017). Bacterial SBP56 identified as a Cu-dependent methanethiol oxidase widely distributed in the biosphere. *ISME J.* 12 145–160. 10.1038/ismej.2017.148 29064480 PMC5739008

[B19] Ferrera I. Sebastian M. Acinas S. G. Gasol J. M. (2015). Prokaryotic functional gene diversity in the sunlit ocean: stumbling in the dark. *Curr. Opin. Microbiol.* 25 33–39. 10.1016/j.mib.2015.03.007 25863027

[B20] Georges A. A. El-Swais H. Craig S. E. Li W. K. Walsh D. A. (2014). Metaproteomic analysis of a winter to spring succession in coastal northwest Atlantic Ocean microbial plankton. *ISME J.* 8 1301–1313. 10.1038/ismej.2013.234 24401863 PMC4030229

[B21] Hawley A. K. Brewer H. M. Norbeck A. D. Paša-Toliæ L. Hallam S. J. (2014). Metaproteomics reveals differential modes of metabolic coupling among ubiquitous oxygen minimum zone microbes. *Proc. Natl. Acad. Sci. U.S.A.* 111 11395–11400. 25053816 10.1073/pnas.1322132111 PMC4128106

[B22] Jiao N. Luo T. Zhang R. Yan W. Lin Y. Johnson Z. I. (2013). Presence of *Prochlorococcus* in the aphotic waters of the western Pacific Ocean. *Biogeosciences* 10 9345–9371. 10.5194/bgd-10-9345-2013

[B23] Karner M. B. DeLong E. F. Karl D. M. (2001). Archaeal dominance in the mesopelagic zone of the Pacific Ocean. *Nature* 409 507–510. 11206545 10.1038/35054051

[B24] Kerou M. Offre P. Valledor L. Abby S. S. Melcher M. Nagler M. (2016). Proteomics and comparative genomics of *Nitrososphaera viennensis* reveal the core genome and adaptations of archaeal ammonia oxidizers. *Proc. Natl. Acad. Sci. U.S.A.* 113 E7937–E7946. 10.1073/pnas.1601212113 27864514 PMC5150414

[B25] Koch H. Lücker S. Albertsen M. Kitzinger K. Herbold C. Spieck E. (2015). Expanded metabolic versatility of ubiquitous nitrite-oxidizing bacteria from the genus *Nitrospira*. *Proc. Natl. Acad. Sci. U.S.A.* 112 11371–11376. 10.1073/pnas.1506533112 26305944 PMC4568715

[B26] Kozlowski J. A. Stieglmeier M. Schleper C. Klotz M. G. Stein L. Y. (2016). Pathways and key intermediates required for obligate aerobic ammonia-dependent chemolithotrophy in bacteria and Thaumarchaeota. *ISME J.* 10 1836–1845. 10.1038/ismej.2016.2 26882267 PMC5029154

[B27] Lindell D. Jaffe J. D. Johnson Z. I. Church G. M. Chisholm S. W. (2005). Photosynthesis genes in marine viruses yield proteins during host infection. *Nature* 438 86–89. 16222247 10.1038/nature04111

[B28] Lochte K. Turley C. (1988). Bacteria and cyanobacteria associated with Phytodetritus in the deep sea. *Nature* 333 67–69.

[B29] Lucker S. Nowka B. Rattei T. Spieck E. Daims H. (2013). The genome of *Nitrospina gracilis* illuminates the metabolism and evolution of the major marine nitrite oxidizer. *Front. Microbiol.* 4:27. 10.3389/fmicb.2013.00027 23439773 PMC3578206

[B30] Lücker S. Wagner M. Maixner F. Pelletier E. Koch H. Vacherie B. (2010). A *Nitrospira* metagenome illuminates the physiology and evolution of globally important nitrite-oxidizing bacteria. *Proc. Natl. Acad. Sci. U.S.A.* 107 13479. 10.1073/pnas.1003860107 20624973 PMC2922143

[B31] Lund M. B. Smith J. M. Francis C. A. (2012). Diversity, abundance and expression of nitrite reductase (nirK)-like genes in marine thaumarchaea. *ISME J.* 6 1966–1977. 10.1038/ismej.2012.40 22592819 PMC3446794

[B32] Martinez-Gomez N. C. Nguyen S. Lidstrom M. E. (2013). Elucidation of the role of the methylene-tetrahydromethanopterin dehydrogenase MtdA in the tetrahydromethanopterin-dependent oxidation pathway in *Methylobacterium extorquens* AM1. *J. Bacteriol.* 195 2359–2367. 10.1128/JB.00029-13 23504017 PMC3650556

[B33] Moore E. K. Nunn B. L. Goodlett D. R. Harvey H. R. (2012). Identifying and tracking proteins through the marine water column: insights into the inputs and preservation mechanisms of protein in sediments. *Geochim. Cosmochim. Acta* 83 324–359. 22711915 10.1016/j.gca.2012.01.002 PMC3375732

[B34] Moran M. A. Buchan A. González J. M. Heidelberg J. F. Whitman W. B. Kiene R. P. (2004). Genome sequence of *Silicibacter pomeroyi* reveals adaptations to the marine environment. *Nature* 432 910–913. 10.1038/nature03170 15602564

[B35] Morris R. M. Nunn B. L. Frazar C. Goodlett D. R. Ting Y. S. Rocap G. (2010). Comparative metaproteomics reveals ocean-scale shifts in microbial nutrient utilization and energy transduction. *ISME J.* 4 673–685. 10.1038/ismej.2010.4 20164862

[B36] Morris R. M. Rappe M. S. Connon S. A. Vergin K. L. Siebold W. A. Carlson C. A. (2002). SAR11 clade dominates ocean surface bacterioplankton communities. *Nature* 420 806–810. 12490947 10.1038/nature01240

[B37] Murudkar C. S. Kodgire P. Krishnamurthy Rao K. (2006). The carboxy terminal domain of Epr, a minor extracellular serine protease, is essential for the swarming motility of *Bacillus subtilis* 168. *FEMS Microbiol. Lett.* 257 24–31. 10.1111/j.1574-6968.2006.00151.x 16553828

[B38] Pachiadaki M. G. Sintes E. Bergauer K. Brown J. M. Record N. R. Swan B. K. (2017). Major role of nitrite-oxidizing bacteria in dark ocean carbon fixation. *Science* 358 1046–1051. 29170234 10.1126/science.aan8260

[B39] Qin W. Amin S. A. Lundeen R. A. Heal K. R. Martens-Habbena W. Turkarslan S. (2017). Stress response of a marine ammonia-oxidizing archaeon informs physiological status of environmental populations. *ISME J.* 12 508–519. 10.1038/ismej.2017.186 29053148 PMC5776466

[B40] Rinke C. Chuvochina M. Mussig A. J. Chaumeil P.-A. Davín A. A. Waite D. W. (2021). A standardized archaeal taxonomy for the Genome Taxonomy Database. *Nat. Microbiol.* 6 946–959. 10.1038/s41564-021-00918-8 34155373

[B41] Saito M. A. Bertrand E. M. Duffy M. E. Gaylord D. A. Held N. A. Hervey W. J. (2019). Progress and challenges in ocean metaproteomics and proposed best practices for data sharing. *J. Proteome Res.* 18 1461–1476. 10.1021/acs.jproteome.8b00761 30702898 PMC7575043

[B42] Salazar G. Cornejo-Castillo F. M. Benitez-Barrios V. Fraile-Nuez E. Alvarez-Salgado X. A. Duarte C. M. (2016). Global diversity and biogeography of deep-sea pelagic prokaryotes. *ISME J.* 10 596–608. 10.1038/ismej.2015.137 26251871 PMC4817678

[B43] Santoro A. E. Buchwald C. McIlvin M. R. Casciotti K. L. (2011). Isotopic signature of N2O produced by marine ammonia-oxidizing archaea. *Science* 333 1282–1285. 21798895 10.1126/science.1208239

[B44] Santoro A. E. Dupont C. L. Richter R. A. Craig M. T. Carini P. McIlvin M. R. (2015). Genomic and proteomic characterization of “*Candidatus Nitrosopelagicus* brevis”: an ammonia-oxidizing archaeon from the open ocean. *Proc. Natl. Acad. Sci. U.S.A.* 112 1173–1178. 10.1073/pnas.1416223112 25587132 PMC4313803

[B45] Sharon I. Tzahor S. Williamson S. Shmoish M. Man-Aharonovich D. Rusch D. B. (2007). Viral photosynthetic reaction center genes and transcripts in the marine environment. *ISME J.* 1 492–501. 10.1038/ismej.2007.67 18043651

[B46] Shi Y. Tyson G. W. Eppley J. M. Delong E. F. (2010). Integrated metatranscriptomic and metagenomic analyses of stratified microbial assemblages in the open ocean. *ISME J.* 5 999–1013. 21151004 10.1038/ismej.2010.189 PMC3131857

[B47] Sowell S. M. Abraham P. E. Shah M. Verberkmoes N. C. Smith D. P. Barofsky D. F. (2011). Environmental proteomics of microbial plankton in a highly productive coastal upwelling system. *ISME J.* 5 856–865. 10.1038/ismej.2010.168 21068774 PMC3105774

[B48] Sowell S. M. Wilhelm L. J. Norbeck A. D. Lipton M. S. Nicora C. D. Barofsky D. F. (2009). Transport functions dominate the SAR11 metaproteome at low-nutrient extremes in the Sargasso Sea. *ISME J.* 3 93–105. 10.1038/ismej.2008.83 18769456

[B49] Sun J. Mausz M. A. Chen Y. Giovannoni S. J. (2019). Microbial trimethylamine metabolism in marine environments. *Environ. Microbiol.* 21 513–520. 10.1111/1462-2920.14461 30370577

[B50] Sun J. Steindler L. Thrash J. C. Halsey K. H. Smith D. P. Carter A. E. (2011). One carbon metabolism in SAR11 pelagic marine bacteria. *PLoS One* 6:e23973. 10.1371/journal.pone.0023973 21886845 PMC3160333

[B51] Sun J. Todd J. D. Thrash J. C. Qian Y. Qian M. C. Temperton B. (2016). The abundant marine bacterium *Pelagibacter* simultaneously catabolizes dimethylsulfoniopropionate to the gases dimethyl sulfide and methanethiol. *Nat. Microbiol.* 1:16065. 10.1038/nmicrobiol.2016.65 27573103

[B52] Sunagawa S. Coelho L. P. Chaffron S. Kultima J. R. Labadie K. Salazar G. (2015). Structure and function of the global ocean microbiome. *Science* 348:1261359. 25999513 10.1126/science.1261359

[B53] Suttle C. A. (2005). Viruses in the sea. *Nature* 437 356–361. 16163346 10.1038/nature04160

[B54] Swan B. K. Martinez-Garcia M. Preston C. M. Sczyrba A. Woyke T. Lamy D. (2011). Potential for chemolithoautotrophy among ubiquitous bacteria lineages in the dark ocean. *Science* 333 1296–1300. 21885783 10.1126/science.1203690

[B55] Tolli J. D. Taylor C. D. (2005). Biological CO oxidation in the Sargasso Sea and in Vineyard Sound, Massachusetts. *Limnol. Oceanogr.* 50 1205–1212. 10.4319/lo.2005.50.4.1205

[B56] Wang D.-Z. Dong H.-P. Xie Z.-X. Dai M.-H. Hong H.-S. (2011). Metaproteomic characterization of dissolved organic matter in the water column of the South China Sea. *Limnol. Oceanogr.* 56 1641–1652. 10.4319/lo.2011.56.5.1641

[B57] Wen B. Du C. Li G. Ghali F. Jones A. R. Käll L. (2015). IPeak: an open source tool to combine results from multiple MS/MS search engines. *Proteomics* 15 2916–2920. 10.1002/pmic.201400208 25951428

[B58] Williams T. J. Long E. Evans F. DeMaere M. Z. Lauro F. M. Raftery M. J. (2012). A metaproteomic assessment of winter and summer bacterioplankton from Antarctic Peninsula coastal surface waters. *ISME J.* 6 1883–1900. 10.1038/ismej.2012.28 22534610 PMC3446797

[B59] Xie Z.-X. Chen F. Zhang S.-F. Wang M.-H. Zhang H. Kong L.-F. (2018). Metaproteomics of marine viral concentrates reveals key viral populations and abundant periplasmic proteins in the oligotrophic deep chlorophyll maximum of the South China Sea. *Environ. Microbiol.* 20 477–491. 10.1111/1462-2920.13937 28925544

[B60] Xu D. Sun P. Zhang Y. Li R. Huang B. Jiao N. (2018). Pigmented microbial eukaryotes fuel the deep sea carbon pool in the tropical Western Pacific Ocean. *Environ. Microbiol.* 20 3811–3824. 10.1111/1462-2920.14396 30159996

[B61] Zhang X. Ning Z. Mayne J. Moore J. I. Li J. Butcher J. (2016). MetaPro-IQ: a universal metaproteomic approach to studying human and mouse gut microbiota. *Microbiome* 4:31. 10.1186/s40168-016-0176-z 27343061 PMC4919841

[B62] Zhang Y. Qin W. Hou L. Zakem E. J. Wan X. Zhao Z. (2020). Nitrifier adaptation to low energy flux controls inventory of reduced nitrogen in the dark ocean. *Proc. Natl. Acad. Sci. U.S.A.* 117 4823–4830. 10.1073/pnas.1912367117 32071230 PMC7060736

[B63] Zhang Y. Zhao Z. Dai M. Jiao N. Herndl G. J. (2014). Drivers shaping the diversity and biogeography of total and active bacterial communities in the South China Sea. *Mol. Ecol.* 23 2260–2274. 10.1111/mec.12739 24684298 PMC4230472

[B64] Zhao Z. Baltar F. Herndl G. J. (2020). Linking extracellular enzymes to phylogeny indicates a predominantly particle-associated lifestyle of deep-sea prokaryotes. *Sci. Adv.* 6:eaaz4354. 32494615 10.1126/sciadv.aaz4354 PMC7159927

