# Supplementary material for: Metaexoproteomics Reveals Microbial Behavior in the Ocean’s Interior
Source: Front Microbiol. 2022 Feb 16;13:749874. doi: 10.3389/fmicb.2022.749874 (PMC8889253; doi:10.3389/fmicb.2022.749874)
Supplement: Supplementary Figure 1 — Scatter plot showing the abundance relationship between phage proteins and host exoproteins. Dots with different colors indicate different host groups. [file Image_1.pdf]

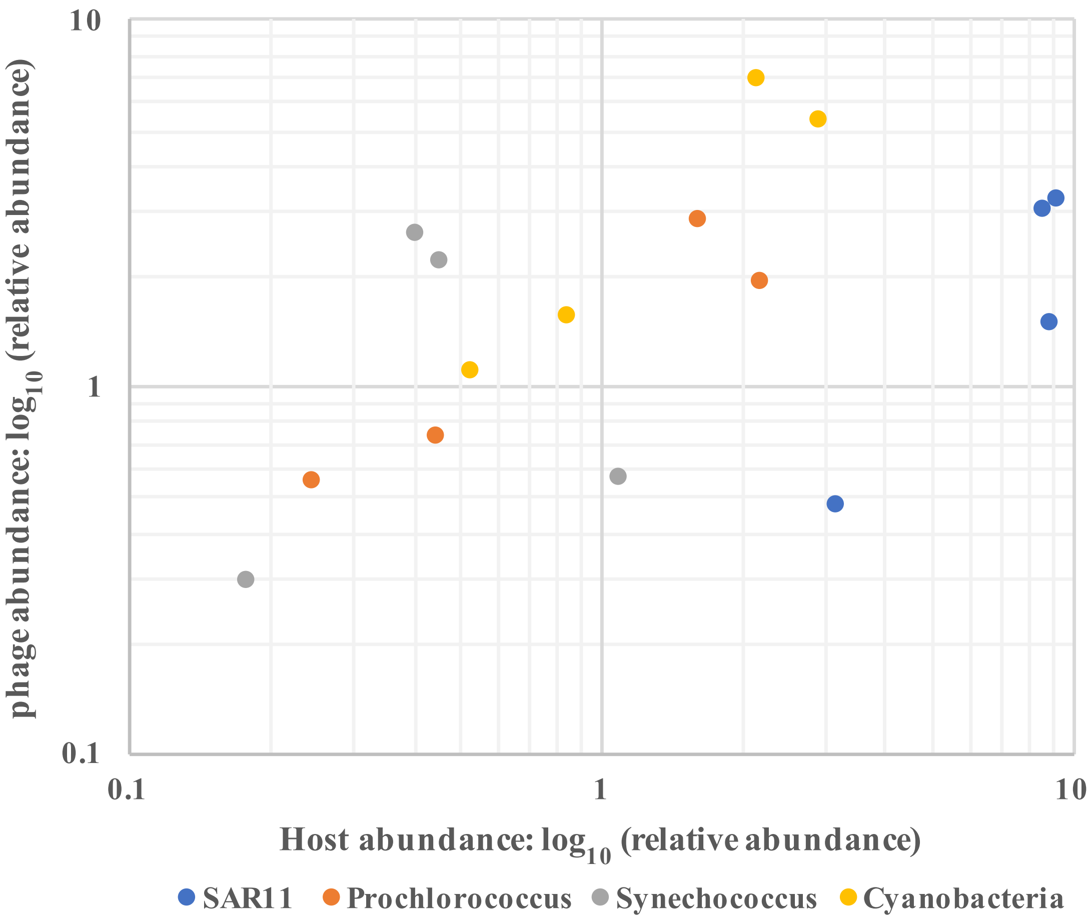

Figure S1. Scatter plot showing the abundance relationship between phage proteins and host exoproteins. Dots with different colors indicate different host groups.
