# Supplementary material for: Metaexoproteomics Reveals Microbial Behavior in the Ocean’s Interior
Source: Front Microbiol. 2022 Feb 16;13:749874. doi: 10.3389/fmicb.2022.749874 (PMC8889253; doi:10.3389/fmicb.2022.749874)
Supplement: Supplementary file 7 [file Table_1.docx]

Supplementary Information

Table S1. List of virus-encoded auxiliary metabolic proteins detected in exoproteomes from the water column of the South China Sea.

| Protein ID | Taxa assignment | KO Definition | COG Description | NCBI Description | DCM_1 | DCM_2 | 200 m | 3000 m |
| --- | --- | --- | --- | --- | --- | --- | --- | --- |
| SEATS-dcm_GL1236487 | Caudovirales, Siphoviridae, putative cyanophage | - | - | putative carbohydrate binding domain containing protein |  | 0.0112 |  |  |
| A_OM-RGC.v1.029522042 | Caudovirales, Siphoviridae, putative pelagiphage | - | - | putative carbohydrate binding domain containing protein |  | 0.0234 |  |  |
| A_OM-RGC.v1.004235908 | Caudovirales, Siphoviridae, uncultured MED phage | - | - | 3-phosphoglycerate kinase |  | 0.0070 |  |  |
| A_OM-RGC.v1.004486274 | Caudovirales, Siphoviridae, uncultured MED phage | - | - | 3-phosphoglycerate kinase | 0.0148 | 0.0072 |  |  |
| A_OM-RGC.v1.004924722 | Caudovirales, Siphoviridae, uncultured MED phage | - | - | 3-phosphoglycerate kinase |  | 0.0037 |  |  |
| A_OM-RGC.v1.001532964 | Caudovirales, uncultured MED phage | - | - | 2,3-dihydro-2,3-dihydroxybenzoate dehydrogenase | 0.0052 |  |  |  |
| A_OM-RGC.v1.002212457 | Caudovirales, uncultured MED phage | - | - | 2,3-dihydro-2,3-dihydroxybenzoate dehydrogenase | 0.0087 | 0.0168 |  |  |
| A_OM-RGC.v1.004807044 | Caudovirales, uncultured MED phage | - | - | 2,3-dihydro-2,3-dihydroxybenzoate dehydrogenase |  | 0.0074 |  |  |
| SS1-5m_GL0841351 | unclassified phages, uncultured MED phage | - | - | outer membrane protein | 0.0052 |  |  |  |
| A_OM-RGC.v1.000195008 | unclassified phages, uncultured MED phage | iron complex outermembrane recepter protein | Outer membrane receptor for Fe3+-dicitrate | putative transporter |  | 0.0027 | 0.0024 |  |
| A_OM-RGC.v1.000232699 | unclassified phages, uncultured MED phage | iron complex outermembrane recepter protein | Outer membrane receptor for Fe3+-dicitrate | putative transporter |  |  | 0.0099 |  |
| A_OM-RGC.v1.000243928 | unclassified phages, uncultured MED phage | iron complex outermembrane recepter protein | Outer membrane receptor for Fe3+-dicitrate | putative transporter |  | 0.0043 |  |  |
| A_OM-RGC.v1.000381243 | unclassified phages, uncultured MED phage | iron complex outermembrane recepter protein | Outer membrane receptor for Fe3+-dicitrate | putative transporter |  |  | 0.0014 |  |
| A_OM-RGC.v1.000438674 | unclassified phages, uncultured MED phage | iron complex outermembrane recepter protein | Outer membrane receptor for Fe3+-dicitrate | putative transporter |  |  | 0.0030 |  |
| A_OM-RGC.v1.000557558 | unclassified phages, uncultured MED phage | - | Outer membrane receptor for Fe3+-dicitrate | putative transporter | 0.0056 |  | 0.0016 |  |
| A_OM-RGC.v1.000790491 | unclassified phages, uncultured MED phage | - | Outer membrane receptor for Fe3+-dicitrate | putative transporter |  |  | 0.0018 | 0.0071 |
| A_OM-RGC.v1.000823945 | unclassified phages, uncultured MED phage | - | Outer membrane receptor for Fe3+-dicitrate | putative transporter |  |  | 0.0072 |  |
| A_OM-RGC.v1.000888042 | unclassified phages, uncultured MED phage | - | Outer membrane receptor for Fe3+-dicitrate | putative transporter |  |  | 0.0018 |  |
| A_OM-RGC.v1.001504187 | unclassified phages, uncultured MED phage | - | Outer membrane receptor for Fe3+-dicitrate | putative transporter |  |  | 0.0022 |  |
| A_OM-RGC.v1.001508376 | unclassified phages, uncultured MED phage | iron complex outermembrane recepter protein | Outer membrane receptor for Fe3+-dicitrate | putative transporter |  |  | 0.0044 |  |
| A_OM-RGC.v1.003694306 | unclassified phages, uncultured MED phage | - | Outer membrane receptor for Fe3+-dicitrate | putative transporter |  |  | 0.0058 |  |
| A_OM-RGC.v1.005530795 | unclassified phages, uncultured MED phage | iron complex outermembrane recepter protein | Outer membrane receptor for Fe3+-dicitrate | putative transporter |  | 0.0077 | 0.0034 |  |
| SEATS-200m_GL0256296 | unclassified phages, uncultured MED phage | - | Outer membrane receptor for Fe3+-dicitrate | putative transporter |  |  | 0.0020 |  |
| SEATS-750m_GL0068792 | unclassified phages, uncultured MED phage | iron complex outermembrane recepter protein | Outer membrane receptor for Fe3+-dicitrate | putative transporter |  |  | 0.0012 |  |
| SEATS-750m_GL0172332 | unclassified phages, uncultured MED phage | iron complex outermembrane recepter protein | Outer membrane receptor for Fe3+-dicitrate | putative transporter |  |  |  | 0.0112 |
| SEATS-750m_GL0188601 | unclassified phages, uncultured MED phage | iron complex outermembrane recepter protein | Outer membrane receptor for Fe3+-dicitrate | putative transporter |  |  | 0.0018 |  |
| SEATS-750m_GL0535695 | unclassified phages, uncultured MED phage | iron complex outermembrane recepter protein | Outer membrane receptor for Fe3+-dicitrate | putative transporter |  |  |  | 0.0143 |
| SS1-200m_GL0194361 | unclassified phages, uncultured MED phage | - | Outer membrane receptor for Fe3+-dicitrate | putative transporter |  |  | 0.0072 |  |
| SS1-200m_GL0518141 | unclassified phages, uncultured MED phage | - | Outer membrane receptor for Fe3+-dicitrate | putative transporter |  |  | 0.0031 |  |
| SS1-3000m_GL0531203 | unclassified phages, uncultured MED phage | - | Outer membrane receptor for Fe3+-dicitrate | putative transporter |  |  |  | 0.0097 |
| SS1-5m_GL0309858 | unclassified phages, uncultured MED phage | iron complex outermembrane recepter protein | Outer membrane receptor for Fe3+-dicitrate | putative transporter |  | 0.0036 |  |  |
| A_OM-RGC.v1.026087748 | Caudovirales, uncultured MED phage | general L-amino acid transport system substrate-binding protein | ABC-type amino acid transport/signal transduction system, periplasmic component/domain | lysine-arginine-ornithine-binding periplasmic protein |  |  | 0.0831 |  |
| A_OM-RGC.v1.015980043 | Caudovirales, uncultured MED phage | chaperonin GroES | Co-chaperonin GroES (HSP10) | hypothetical protein | 0.0066 | 0.0063 |  |  |
| SS1-200m_GL2455896 | Caudovirales, uncultured MED phage | chaperonin GroES | Co-chaperonin GroES (HSP10) | hypothetical protein | 0.0118 |  |  |  |
| A_OM-RGC.v1.000418627 | unclassified uncultured deep Atlantic vSAG | chaperonin GroES | Co-chaperonin GroES (HSP10) | hypothetical protein |  | 0.0050 |  |  |
| A_OM-RGC.v1.001165480 | unclassified uncultured deep Atlantic vSAG | chaperonin GroES | Co-chaperonin GroES (HSP10) | hypothetical protein | 0.0071 | 0.0091 |  |  |
| SEATS-5m_GL2233436 | unclassified uncultured deep Atlantic vSAG | chaperonin GroES | Co-chaperonin GroES (HSP10) | hypothetical protein | 0.0099 |  |  |  |
| A_OM-RGC.v1.002063403 | unclassified uncultured surface MED vSAG | chaperonin GroES | Co-chaperonin GroES (HSP10) | hypothetical protein | 0.0028 |  |  |  |
| A_OM-RGC.v1.002790841 | unclassified uncultured surface MED vSAG | chaperonin GroES | Co-chaperonin GroES (HSP10) | hypothetical protein | 0.0062 |  |  |  |
| A_OM-RGC.v1.000771239 | Phycodnaviridae, Chrysochromulina ericina virus | heat shock 70kDa protein 1/2/6/8 | Molecular chaperone DnaK (HSP70) | Hsp70 protein |  |  |  | 0.0071 |
| A_OM-RGC.v1.000849208 | Phycodnaviridae, Prasinovirus | heat shock 70kDa protein 1/2/6/8 | Molecular chaperone DnaK (HSP70) | heat shock protein 70 |  |  | 0.0091 |  |
| SEATS-750m_GL1558607 | Caudovirales, Myoviridae, Prochlorococcus phage | photosystem II P680 reaction center D1 protein [EC:1.10.3.9] | - | photosystem II D1 protein |  |  | 0.0240 |  |
| A_OM-RGC.v1.004753653 | Caudovirales, Myoviridae, Synechococcus phage | photosystem II P680 reaction center D2 protein [EC:1.10.3.9] | - | photosystem II D2 protein |  |  |  | 0.0130 |
| A_OM-RGC.v1.026033837 | Caudovirales, Podoviridae, Prochlorococcus phage | photosystem II P680 reaction center D1 protein [EC:1.10.3.9] | - | photosystem II reaction center protein PsbA/D1 | 0.0097 |  |  |  |
| A_OM-RGC.v1.020633535 | unclassified phages, uncultured MED phage | photosystem II P680 reaction center D1 protein [EC:1.10.3.9] | - | psbA photosystem II, DI subunit | 0.0076 |  |  |  |
| SEATS-5m_GL3774307 | unclassified viruses | photosystem II P680 reaction center D1 protein [EC:1.10.3.9] | - | photosystem II protein D1, partial |  | 0.0495 |  |  |
| SS1-dcm_GL1282960 | Caudovirales, Myoviridae, Synechococcus phage | thymidylate synthase (FAD) [EC:2.1.1.148] | Thymidylate synthase ThyX | thymidylate synthase |  | 0.0390 |  |  |
